# Chorea

**Published:** 1886-09

**Authors:** 


### *CHOREA.*

The discussion of this malady is so frequent in the journals and the treatment and results of treatment are so varied, that one inexperienced in its treatment is liable to ask if there is any one form of treatment more reliable than all others. To show some of these varieties we quote from the Fort Wayne *Medical Journal* the following resumé of forty forms of treatment:

"Spencer M. Free, M. D., Baltimore, (*Maryland Medical Journal*, April 24, 1886): After discussing the causes, Dr. Free says of treatment, that drugs have been employed extensively as to number and dosage. With few exceptions they are valueless.

"The first to be recommended is, as far as possible, fresh air, outdoor exercise, avoidance of excitement, proper bathing, plain and nourishing food. If the case is severe, rest in bed may be of advantage.

"If a cause is discoverable, as worms, decayed teeth, nasal catarrh, etc., remove it. Without a careful search we have come upon thirty-nine forms of treatment.

"*Strychnia* has its warm advocates. Trousseau probably is its best exponent. He uses a solution of the sulphate. He gives it in a dose of $\frac{1}{300}$ of a grain t. i. d., gradually increasing the amount to 1 grain per day, He cautions concerning the great danger, and enjoins care and watchfulness.

"West and Bouchet oppose its use on account of the danger, as a number of deaths have been produced by it.

"In all anæmic cases tonics are called for. Iron, in some form, is preferred by nearly all writers. Radcliff uses the iodide, J. Lewis Smith the ammonio-citrate. The mur. tinct. is generally used. The emulsion of cod-liver oil with the hypophosphites of lime and soda has been used with good effect.

"Dr. Young, of Philadelphia, prefers cimicifuga.

"Dr. West, sulphate of zinc.

"Drs. Steiner and Hufland, oxide of zinc.

"Dr. Wier Mitchell, salicylate of soda, especially in cases of rheumatic diathesis.

"Dr. J. H. Carstens, propylamine.

"Dr. Goodheart, rest

"Drs. C. L. Dana, Mills, Webber, Rockwell and Beard, galvanization of brain.

"Drs. Baunis and Burnheim regard hypnotism a specific. Only a few seances are necessary.

"Applications of cold to the spine, by means of the wet pack, a jet of cold water, or the ether spray have been used quite extensively and with good effect. Some advocate the cold bath, or cold shower-bath. I have used the cold wet pack in several cases with excellent results. I follow the packing by rubbing with olive oil. These cold applications are used in conjunction with internal medication.

"The one remedy, which is the main reliance of the great majority of practitioners, is arsenic. It is usually given in the form of Fowler's solution, in a gradually increasing dose. Of those who rely chiefly upon it are Smith (J. Lewis), Leese, Rayer, Martin, Gregory, Latter, Babbington, Hughes, Begbie, Romberg, Dieudonne, Barthez, Aran, Edes, Hammond and Seguin.

"Dr. Hammond strongly advocates its use hypodermically.

"Dr. Gelle says that it fails in nervous and sanguine patients.

"Drs. Romberg and Bourguignon agree with him.

"In a series of cases reported by Dr. Chapin, of New York, treated entirely by arsenic, in which he compares his results with those obtained by Drs. Gray and Tuckwell, who used the expectant plan, the result was twelve days in favor of the arsenic treatment.

"Some few are doubtful as to the value of any treatment, but the results obtained show a shortening of the diseases by judicious management and medication."

In a paper read before the Medical Society, of the State of New York, in 1882, by Dr. E. C. Seguin, of New York, on "The Efficient Dosage of Certain Remedies Used in the Treatment of Nervous Diseases," he says: "My own experience is in substantial accord with that of Radcliffe and of Begbie, in that I have almost never known arsenic to fail to cure chorea, and often very quickly. I have almost always given Fowler's solution by the stomach, in doses ranging from three to thirty drops three times a day.

"It is exhibited largely diluted with water, usually half a tumberful, or from three to four ounces, and given after food, although I am now inclined to think that the importance of this latter caution has been over-estimated, and is not as great as is that of proper dilution. In children who are delicate, and in sensitive adults, I sometimes commence with a dose of two drops after each meal; more usually with a dose of five drops. Each day I add one drop, thus: On the first day the patient takes five drops, three times a day; on the second day, six drops, three times a day; the third, seven drops, and so on.

"Usually when a dose of from ten to fourteen drops, three times a day, is attained, some arsenical symptoms appear; these are diarrhœa, nausea, vomiting, anorexia, redness and puffiness about the eyes; one of these symptoms or any combination of them. They are not serious, and during their prevalence I have never found albumen in the urine. My practice formerly was to go back to smaller doses when this condition developed, and to again increase; but, in the last two years, I find it more advantageous to withhold all arsenic for forty-eight hours, and then resume at the last dose and begin a further increase. A remarkable tolerance is now shown by most patients, even by young children, and doses of twenty, twenty-five and even thirty drops, thrice a day, may be reached without a renewal of these symptoms. * * * * * *

"I have been taught by experience not to expect amelioration of the choreic movements until the toxic effect of arsenic are evident; and in old, or relapsing cases, not until the second period of toxic symptoms. I nearly always combine rest in bed, in some cases, with the arsenical treatment, and if the patient be wakeful or nervous at night, an occassional bed-time dose of chloral hydrate *per os*, or by enema is given.

"Simple acute chorea may often be cured in two weeks' time by this plan, the positive contradiction to the too prevalent notions of self-limitation of chorea and skepticism as to the efficacy of drugs in this disease.

"As regards possible ill-effects from this arsenical treatment, I would repeat that, though I have examined the urine of many patients at the periods of saturation by arsenic, I have never found albumen or tube-casts. Stomatitis I have seen once, in the case of a physician's son, whose chorea was very rapidly and permanently cured by moderate doses (about eighteen drops three times daily.) Cutaneous symptoms I have never met with, and the gastro-intestinal irritation has never been serious or permanent. My experience with the hypodermic injection of Fowler's solution is more limited. I have made use of it in chronic chorea, and in local choreiform affections, and I would agree with Dr. Hammond, in his statement that we relatively obtain less constitutional disturbance and more curative effect from this method."

